# Mending the Gap: A Systematic Review and Meta-Analysis of Mesh Reinforcement for Abdominal Wall Closure in Abdominal Aortic Aneurysm (AAA) Repair

**DOI:** 10.7759/cureus.93557

**Published:** 2025-09-30

**Authors:** Sara Ahmad, Mohammad U Ahmad, Boroumand Zeidaabadi, Muhammed Siddiqui

**Affiliations:** 1 Department of Surgery, Queen's Hospital, Romford, GBR; 2 Department of Surgery, Manchester University NHS Foundation Trust, Manchester, GBR; 3 School of Medicine, Imperial College London, London, GBR; 4 Department of Surgery, South Tyneside and Sunderland NHS Foundation Trust, Sunderland, GBR

**Keywords:** abdominal aortic aneurysms, hernia mesh, incision hernia, management of abdominal aortic aneurysms, mesh repair, vascular surgery

## Abstract

Incisional hernias are a common complication following abdominal aortic aneurysm (AAA) repair, leading to significant morbidity, higher healthcare costs, and reduced quality of life. While mesh reinforcement is widely used in abdominal surgery, its role in AAA repair remains uncertain due to limited evidence from randomised controlled trials (RCTs) and ongoing concerns about postoperative complications such as infection and seroma formation. A systematic review and meta-analysis of RCTs evaluated the effectiveness of mesh for primary abdominal wall closure post-AAA repair. Comprehensive searches were conducted in MEDLINE, Cochrane, Cumulative Index of Nursing and Allied Health Literature (CINAHL), National Health Service (NHS) National Electronic Library for Health, and PubMed, with no restrictions on date, language, or publication status. Primary outcomes included incisional hernia rates, reoperation rates, infection rates, and seroma formation rates. Statistical analyses utilised random-effects models, reporting odds ratios (OR) with 95% confidence intervals (CI). Heterogeneity was assessed using the I² statistic. Mesh reinforcement significantly reduced the risk of incisional hernias by 87% (OR: 0.13; 95% CI: 0.04-0.46; p=0.001). However, seroma formation was over seven times more likely in the mesh group (OR: 7.13; 95% CI: 1.74-29.28; p=0.006). Infection rates were comparable (OR: 2.59; 95% CI: 0.30-22.24; p=0.39), while reoperation rates were lower but not statistically significant (OR: 0.72; 95% CI: 0.21-2.43; p=0.6). Mesh reinforcement provides a clear benefit in significantly reducing incisional hernias post-AAA repair but increases seroma risk. Surgical decision-making should weigh these benefits against the potential harms. Future research should not only assess long-term outcomes but also establish the optimal mesh material, placement technique, and patient selection criteria to maximise benefits while minimising complications.

## Introduction and background

Rationale

Abdominal aortic aneurysm (AAA) is a critical health concern with potentially life-threatening consequences. In the United Kingdom, the National Health Service (NHS) AAA screening program has reported a prevalence of 1.34%, highlighting its significance as a public health issue [1]. Globally, among individuals aged 30-79 years, AAA affects approximately 0.92% of the population, equating to 35.12 million people [2]. An ageing population, combined with increased screening, is expected to drive further rises in detection. This means that the number of patients requiring AAA repair and thus incisional hernias is expected to increase [3]. Surgery remains the only curative option for AAA, and when undertaken with the open approach, a large laparotomy incision is made to access the aneurysm. One of the most significant complications associated with this procedure is the development of an incisional hernia. The incidence of incisional hernias following AAA repair has been reported to be as high as 46.5% [4]. These hernias carry considerable morbidity and mortality and place an economic burden on healthcare systems through extended follow-up, reoperations, and prolonged hospital stays [5]. Incisional hernias not only pose immediate threats but can also lead to long-term complications affecting quality of life. The potential for bowel obstruction, strangulation, and subsequent compromised perfusion to abdominal organs emphasises the gravity of this condition. Moreover, the recurrence of hernias after repair surgeries further exacerbates the burden on patients and healthcare systems [6]. Patients undergoing open AAA repair frequently present with comorbidities such as hypertension, diabetes, and chronic obstructive pulmonary disease. These conditions may increase vulnerability to postoperative complications, including hernia formation. Despite advances in operative techniques and perioperative care, the best method of abdominal wall closure in this high-risk group remains debated [7]. Various techniques have been used to close the abdominal wall after AAA repair, including primary suture closure and mesh reinforcement. While mesh has reduced hernia rates in other abdominal procedures, its use in AAA repair has long been contentious, and at the time, no closure technique had been shown to be clearly superior [8]. Interest in prophylactic mesh placement is growing, but adoption has been limited due to concerns over infection, mesh integration, and long-term outcomes [9]. Clarifying risk factors and preventive strategies for incisional hernia is essential to reduce postoperative morbidity and support recovery in vascular patients. Although mesh has shown promise in other abdominal surgeries, its role in AAA repair remains uncertain. The technique remains a divisive issue, with no closure method yet proven superior. Clear, evidence-based guidelines are needed to inform surgical decision-making, optimise mesh use, and guide patient selection.

Objective

In response to the lack of consensus on hernia prevention strategies post-AAA repair, this study assesses the impact of mesh-reinforced primary closure on incisional hernia rates.

## Review

Method

Protocol and Registration

This review protocol was registered with the International Prospective Register of Systematic Reviews (PROSPERO) (registration ID: CRD42022360050).

Search Strategy

**Figure 1:**
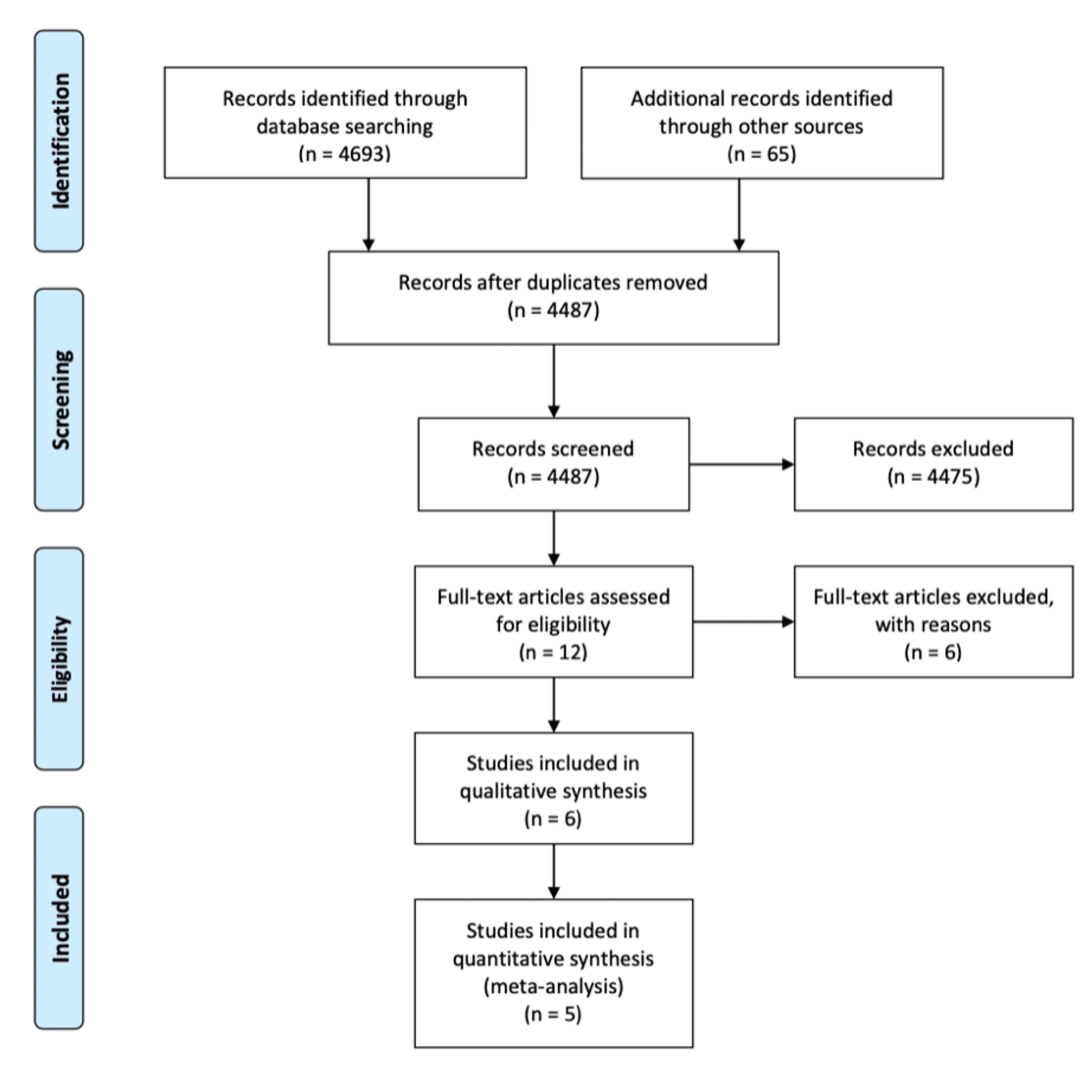
PRISMA flow diagram PRISMA: Preferred Reporting Items for Systematic Reviews and Meta-Analyses

A comprehensive literature search was conducted using the MEDLINE, Cochrane, Cumulative Index of Nursing and Allied Health Literature (CINAHL), NHS National Electronic Library for Health, and PubMed databases. The search was performed between September 15, 2024, and July 2025. No date, language, or publication restrictions were applied. The search strategy was designed using relevant keywords and subject headings (shown in Table 1), related to AAA repair, mesh reinforcement, and incisional hernias. The search strategy combined free-text keywords and Medical Subject Headings (MeSH) using Boolean operators (AND, OR) to optimise sensitivity and specificity. The strategy was tailored to the indexing systems of each database (e.g., MEDLINE, Cochrane, CINAHL, NHS National Electronic Library for Health, and PubMed). Where applicable, search filters and truncation symbols were adapted to fit the syntax of individual platforms. Additionally, the reference lists of included articles and relevant reviews were screened manually to identify any further eligible studies. Figure 1 presents the literature search process as outlined by the Preferred Reporting Items for Systematic Reviews and Meta-Analyses (PRISMA) guidelines.

**Table 1:** Search strategy used for the systematic review

Search domain	Keywords and Boolean terms
Aortic aneurysm and surgical repair	Aortic OR Aorta OR Abdominal Aortic OR Abdominal Aorta OR Aortic Aneurysm OR Aorta Aneurysm OR Abdominal Aortic Aneurysm OR Abdominal Aorta Aneurysm OR Aortic Surgery OR Aorta Surgery OR Abdominal Aortic Surgery OR Abdominal Aorta Surgery OR Aortic Aneurysm Surgery OR Aorta Aneurysm Surgery OR Abdominal Aortic Aneurysm Surgery OR Abdominal Aorta Aneurysm Surgery OR Aortic Repair OR Aorta Repair OR Abdominal Aortic Repair OR Abdominal Aorta Repair OR Aortic Aneurysm Repair OR Aorta Aneurysm Repair OR Abdominal Aortic Aneurysm Repair OR Abdominal Aorta Aneurysm Repair OR Aortic Repair Surgery OR Aorta Repair Surgery OR Abdominal Aortic Repair Surgery OR Abdominal Aorta Repair Surgery OR Aortic Aneurysm Repair Surgery OR Aorta Aneurysm Repair Surgery OR Abdominal Aortic Aneurysm Repair Surgery OR Abdominal Aorta Aneurysm Repair Surgery
Mesh reinforcement and hernia terminology	Mesh OR Mesh Repair OR Primary Mesh Repair OR Prophylactic Mesh Repair OR Mesh Closure OR Primary Mesh Closure OR Mesh Primary Closure OR Biological Mesh OR Biological Mesh Repair OR Primary Biological Mesh Repair OR Prophylactic Biological Mesh Repair OR Biological Mesh Closure OR Primary Biological Mesh Closure OR Biological Mesh Primary Closure OR Non-Biological Mesh OR Non-Biological Mesh Repair OR Primary Non-Biological Mesh Repair OR Prophylactic Non-Biological Mesh Repair OR Non-Biological Mesh Closure OR Primary Non-Biological Mesh Closure OR Non-Biological Mesh Primary Closure OR Hernia OR Incisional Hernia OR Incidental Hernia OR Abdominal Hernia OR Abdominal Wall Hernia OR Abdominal Muscle Hernia OR Abdominal Cavity Hernia OR Incisional Breach OR Incidental Breach OR Abdominal Breach OR Abdominal Wall Breach OR Abdominal Muscle Breach OR Abdominal Cavity Breach OR Incisional Defect OR Incidental Defect OR Abdominal Defect OR Abdominal Wall Defect OR Abdominal Muscle Defect OR Abdominal Cavity Defect

Outcome Measures

The primary outcome was the incidence of incisional hernias following AAA repair surgery. Secondary variables include reoperation rates, infection rates, and length of operation.

Intervention

The intervention was the use of mesh reinforcement for abdominal wall closure following AAA repair surgery.

Comparator

The comparator was no mesh or primary suture closure of the abdominal wall following AAA repair surgery.

Hypotheses and Types of Outcome Measures

The hypothesis is that there is no difference in the use of mesh for abdominal wall closure for incisional hernias compared to primary suture closure.

Statistical Analysis

Statistical analysis was conducted using Review Manager (RevMan; The Cochrane Collaboration, London, UK). Odds ratios (ORs) with 95% confidence intervals (CIs) were calculated for the primary outcome using a random-effects model with the Mantel-Haenszel method [10]. Heterogeneity was assessed using the I² statistic, with values >50% indicating substantial heterogeneity. A random-effects model was chosen due to suspected clinical heterogeneity, and potential sources of heterogeneity were explored when present. Forest plots were generated to visually display effect estimates and heterogeneity.

Study Selection

Four thousand six hundred ninety-three records were identified through database searching, with an additional 65 records identified through other sources. Four thousand four hundred eighty-seven records were screened for relevance, and from those, 4475 were excluded. After assessing for eligibility, we were left with five, and this is represented in Figure 1. The publication year of the studies sampled ranged from 2010 to 2022. Records were removed if they were considered unrelated to our hypothesis. No data was used from any grey or unpublished studies. All randomised controlled trials (RCTs) that compared the use of mesh reinforcement with primary suture closure of the abdominal wall following AAA repair were included. If enough data were available from RCTs, other study types were excluded. There were no regional or date restrictions on any articles. Five studies met the inclusion criteria, encompassing diverse European settings (Greece, the United Kingdom, Belgium, Germany, and the Netherlands). This is demonstrated in Table 2.

**Table 2:** Studies considered This table shows the names and authors of the studies considered and used.

Study number	Title of the study	Authors
1	A comparative study of sutured versus bovine pericardium mesh abdominal closure after open abdominal aortic aneurysm repair	Bali C et al. [11]
2	Randomized clinical trial of mesh versus sutured wound closure after open abdominal aortic aneurysm surgery	Beviset al. [12]
3	Prevention of incisional hernias by prophylactic mesh-augmented reinforcement of midline laparotomies for abdominal aortic aneurysm treatment: five-year follow-up of a randomized controlled trial	Dewulfet al. [13]
4	Abdominal incision defect following AAA-surgery (AIDA): 2-year results of prophylactic onlay-mesh augmentation in a multicentre, double-blind, randomised controlled trial	Honiget al. [14]
5	Prevention of incisional hernia with prophylactic onlay and sublay mesh reinforcement versus primary suture only in midline laparotomies (PRIMA): 2-year follow-up of a multicentre, double-blind, randomised controlled trial	Jairam et al. [15]

Population

The study population included all patients undergoing AAA repair surgery with mesh reinforcement or primary suture closure of the abdominal wall (no mesh). Inclusion criteria from the studies include patients who have been listed for an elective open AAA repair. Patient exclusion criteria included previous surgeries, previous incisional hernias, and if patients were on specific medications, such as steroids and immunosuppression drugs. These drugs were included in the exclusion criteria to eliminate interference with study results. Previous surgeries were included as an exclusion criterion as a person's immune system, metabolism, and general health may be affected by recent surgery, which could skew the study's findings or raise the risk of problems. Due to the unknown and potentially negative consequences of the intervention on the foetus, pregnant women are frequently excluded from clinical trials, giving priority to the mother's and foetus's health. More details on the specifics and complete inclusion and exclusion criteria can be found below in Table 3*.*

**Table 3:** Study types and characteristics, with follow-up periods This table shows the follow-up periods with the participants for each of the studies as well as the study characteristics. The studies have been numbered, and they are referenced below. AAA: abdominal aortic aneurysm; BMI: body mass index; ASA: American Society of Anesthesiologists

Study number	Year	Sample size	Mean age	Country	Inclusion criteria	Exclusion criteria	Follow-up period
1 [11]	2015	40	74.3	Greece	Patients listed for elective open AAA repair between September 2007 and March 2009	Previous abdominal surgery or use of steroids/immunosuppressants	Three years
2 [12]	2010	85	73	United Kingdom	Consecutive patients listed for elective open infrarenal AAA repair (November 2003 to March 2007)	None specified	One month, six months, one year, and then annually up to three years
3 [13]	2022	114	72.1	Belgium	Adults planned for elective AAA via midline laparotomy	Emergency surgery, prior midline mesh, ASA >4, unavailability of abdominal wall surgeon	Five years
4 [14]	2022	104	69.8	Germany	Patients ≥18 years undergoing elective AAA repair via midline incision	Prior midline laparotomy, emergency AAA surgery, limited life expectancy, immunosuppression, coagulopathy, chemo- and radiotherapy, other trials, pregnancy, social/mental limitations	Two years
5 [15]	2017	480	64.5	Austria, Germany, Netherlands	Adults ≥18 years undergoing elective midline laparotomy for AAA or BMI ≥27	Emergency surgery, prior incisional hernia, other trials, limited life expectancy, pregnancy, recent immunosuppression, bovine allergy	Two years

Data Extraction and Quality Assessment

Two investigators independently screened the literature and extracted data according to the inclusion and exclusion criteria. Any disagreement between investigators was resolved by a third investigator. The following data were collected from the studies: author's name, year of publication, study location, basic information of subjects (age, biological sex, the proportion of patients undergoing AAA repair), and outcome data (incisional hernia rates, reoperation rates, infection rates, and length of operation). Risk of bias in included studies was assessed using the Cochrane Risk of Bias tool [16]. The following domains were assessed: random sequence generation, selection bias and allocation concealment, participant and personnel blinding, outcome assessment blinding, incomplete outcome data bias, and selective reporting (Table 4).

**Table 4:** Risk of bias chart This table represents the methodological quality assessment results. The following domains were assessed: random sequence generation, selection bias and allocation concealment, participant and personnel blinding, outcome assessment blinding, incomplete outcome data bias, and selective reporting. For study allocations, please refer to Table 1.

Study	Random sequence	Allocation concealment	Blinding participants	Blinding outcome	Incomplete data	Selective reporting
1 [11]	Low risk	Unclear risk	High risk	High risk	Low risk	Low risk
2 [12]	Low risk	Low risk	Unclear risk	High risk	Low risk	Low risk
3 [13]	Low risk	Low risk	Low risk	Low risk	Unclear risk	Low risk
4 [14]	Low risk	Unclear risk	Low risk	Low risk	Unclear risk	Unclear risk
5 [15]	Low risk	Low risk	Low risk	Low risk	Unclear risk	Low risk

Study Characteristics and Study Types

All five studies were published in English. All studies were carried out in European centres, with study 1 being carried out in Greek centres, study 2 in the United Kingdom, study 3 in Belgium, study 4 in Germany, and study 5 in Germany, Austria, and the Netherlands. The studies with their corresponding follow-up periods and characteristics can be found in Table 3.

Participants

Mesh: Patient characteristics for the mesh group are defined in Table 5. It includes the number of patients in the study, further defining how many were male and female, as well as the mean age and intervention type.

**Table 5:** Patient characteristics (mesh group) This table outlines the grouped patient characteristics from the studies analysed. These are of the patients in the mesh group.

Study number	Number of patients	Age (mean)	Male	Female	Intervention type
1 [11]	20	75	18	2	Suture fascia closure reinforced by an onlay mesh implantation (placement of bovine pericardium mesh above the fascia)
2 [12]	40	74	34	6	Prophylactic placement of polypropylene mesh in the preperitoneal plane
3 [13]	56	72.3	54	2	Prophylactic retrorectus mesh reinforcement with a large-pore polypropylene mesh (Ultrapro (Ethicon Inc., Johnson & Johnson, Somerville, New Jersey, United States), width 7.5 cm)
4 [14]	34 in group 2	70.5	33	1	Long-term absorbable suture and onlay mesh reinforcement (group 2, MonoPlus (Aesculap AG, Tuttlingen, Germany) and mesh)
5 [15]	373	64.3	224	149	188 patients were allocated onlay mesh reinforcement (18 no mesh), and 185 were assigned sublay mesh reinforcement (27 no mesh)

No mesh: The same as above was done for the no mesh group. All relevant information is presented and can be found in Table 6.

**Table 6:** Patient characteristics (no mesh group) This table outlines the grouped patient characteristics from the studies analysed. These are of the patients in the no mesh group. AAA: abdominal aortic aneurysm

Study number	Number of patients	Age (mean)	Male	Female	Intervention type
1 [11]	20	75	18	2	Suture fascia closure
2 [12]	45	72	43	2	Routine abdominal mass closure after AAA repair
3 [13]	58	71.9	51	7	Primary closure of their midline laparotomy after open AAA repair
4 [14]	70 (35 in group 1 and 35 in group 3)	68.5	61 (32 in group 1 and 29 in group 3)	9 (3 in group 1 and 6 in group 3)	Fascial closure with long-term absorbable suture (group 1, MonoPlus (Aesculap AG, Tuttlingen, Germany)) and/or extra-long-term absorbable, more flexible, and elastic suture (group 3, MonoMax (Aesculap AG, Tuttlingen, Germany))
5 [15]	107	65.2	68	39	Primary suture only

Results/Data

A forest plot was generated to visualise the variation in infection rates between the mesh and no mesh groups across the included studies. Each study is represented by a square, with the size of the square corresponding to the weight of the study in the meta-analysis. The horizontal lines extending from each square represent the 95% CI for the effect estimate.

Quantitative Synthesis of Incisional Hernia Rates

**Figure 2:**
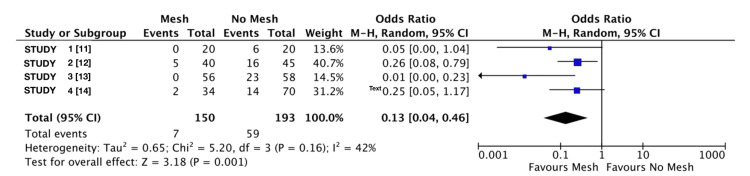
Incisional hernia rate forest plot This forest plot represents the data obtained from the search on associations between incisional hernias and both groups using a mesh and without.

Data have been included from multiple studies investigating the impact of mesh use on incisional hernia rates, with four studies represented in the forest plot (Figure 2). In total, 323 patients (150 in the mesh group and 173 in the no mesh group) were analysed. The results demonstrated that the use of mesh significantly reduced the odds of developing incisional hernias. Patients who underwent mesh repair were approximately 87% less likely to develop incisional hernias compared to those in the no mesh group (95% CI: 54% to 96% lower odds; p=0.001). The overall OR across these four studies, as depicted, was 0.13 (95% CI: 0.04 to 0.46; p=0.001). The analysis of variability revealed moderate heterogeneity, with 42% of the variability attributed to factors beyond random chance (p=0.16) [11-14].

Quantitative Synthesis of Seroma Rates

**Figure 3:**
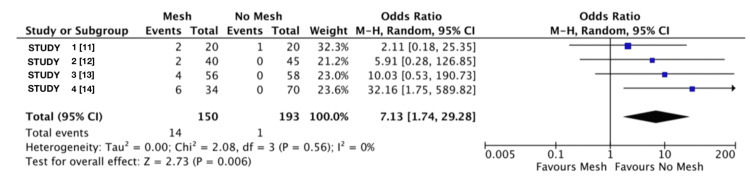
Seroma rate forest plot This forest plot represents the data obtained from the search on associations between seromas and both groups using a mesh and without.

As shown in Figure 3, the use of mesh was associated with a significantly increased likelihood of seroma formation. The pooled analysis estimated that patients undergoing mesh repair were over seven times more likely to develop seromas compared to those in the no mesh group (overall OR: 7.13; 95% CI: 1.74 to 29.28; p=0.006). The pooled analysis showed no significant heterogeneity between the studies (Tau²=0.00; Chi²=2.8; df=3; p=0.56; I²=0%) [11-14].

Quantitative Synthesis of Reoperation Rates

**Figure 4:**
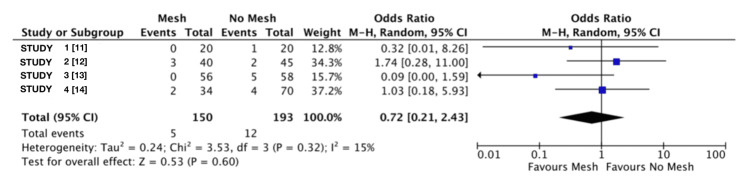
Reoperation rate forest plot This forest plot represents the data obtained from the search on associations between reoperation rates and both groups using a mesh and without.

As shown in Figure 4, the overall findings indicated no statistically significant difference in the likelihood of reoperation between the mesh and no mesh groups. The pooled analysis estimated that the odds of requiring a reoperation were approximately 28% lower in the mesh group, but this result was not statistically significant (overall OR: 0.72; 95% CI: 0.21 to 2.43; p=0.6). The analysis of heterogeneity indicated low variability between studies, with an I² value of 15% (Tau²=0.24; Chi²=3.53; df=3; p=0.32) [11-14].

Quantitative Synthesis of Infection Rates

**Figure 5:**
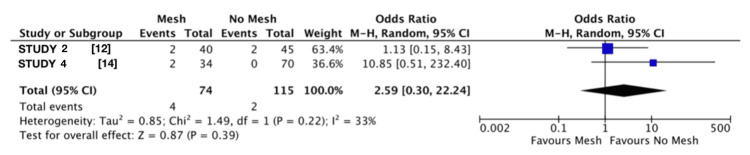
Infection rate forest plot This forest plot represents the data obtained from the search on associations between infection rates and both groups using a mesh and without.

The findings indicated no statistically significant difference in infection rates between the mesh and no mesh groups (Figure 5). The pooled analysis estimated that the odds of infection were 2.59 times higher in the mesh group, but this result was not statistically significant (95% CI: 0.30 to 22.24; p=0.39). The heterogeneity analysis indicated moderate variability between the studies, with an I² value of 33% (Tau²=0.85; Chi²=1.49; df=1; p=0.22) [12,14].

Discussion

Main Findings

In the quantitative synthesis of incisional hernia rates, the calculated heterogeneity may reflect differences in study design, surgical techniques, and patient populations. Although the results indicate a consistent benefit from the use of mesh, it is important to acknowledge that some studies reported very low or no hernia events in the mesh group, which could influence the pooled effect estimate. Furthermore, the forest plot represents only a subset of the studies included in the broader meta-analysis, selected for their comprehensive reporting and relevance to the analysis. There is strong evidence supporting the effectiveness of mesh in reducing the incidence of incisional hernias. However, the moderate heterogeneity observed and variation in study methodologies reinforce the need for further high-quality RCTs to validate these findings and assess long-term outcomes.

The lack of heterogeneity in the analysis of seroma incidence suggests that variation across the included studies is minimal. The test for overall effect (Z=2.73; p=0.006) indicates that the increased odds of seroma formation associated with mesh use are unlikely due to chance. However, the wide confidence intervals observed in individual studies highlight the need for cautious interpretation, as the results may be influenced by small sample sizes and the relatively low number of reported seroma events.

Regarding reoperation rates, the analysis revealed low heterogeneity, suggesting that differences across studies are mostly attributable to random variation rather than systematic discrepancies. The test for overall effect (Z=0.53; p=0.6) supports the conclusion that there is no statistically significant association between mesh use and reoperation rates. Nonetheless, wide confidence intervals observed in individual studies indicate uncertainty in the effect estimate, likely influenced by the small number of reoperation events.

Although the level of heterogeneity in the quantitative synthesis of infection rates is not statistically significant, it implies some differences between studies that could stem from variations in patient populations, surgical techniques, or follow-up protocols. The test for overall effect (Z=0.87; p=0.39) confirms that the observed difference in infection rates between the mesh and no mesh groups is not statistically significant. The wide confidence intervals in both individual studies and the pooled estimate reflect uncertainty in the effect size, likely driven by the small number of infection events. One way to explore this further would be to carry out a sensitivity analysis, for example, by excluding studies that carry a high risk of bias or report only a small number of events, to see if the main finding remains consistent.

This meta-analysis of RCTs indicates a significant reduction in the incidence of incisional hernias when mesh is used for primary closure of the abdominal wall following AAA repair surgery. This finding aligns with prior studies [17], which have also demonstrated the efficacy of mesh reinforcement in preventing hernia formation. Notably, the results also suggest that increased seroma formation is a relevant complication. The limited number of available RCTs and the potential for bias emphasise the need for further research to validate these findings and to determine the optimal mesh type and placement technique.

Summary and Appraisal of Evidence

The evidence synthesised in this meta-analysis offers valuable insights into the efficacy of mesh reinforcement in reducing incisional hernia rates following AAA repair. The inclusion of RCTs facilitates a robust analysis of the available data, contributing to a more comprehensive understanding of both the benefits and limitations of mesh techniques in this clinical setting. However, the quality of the evidence is affected by inherent limitations in the included studies, such as small sample sizes, variations in surgical technique, and potential sources of bias.

Importance of This Study

An important clinical question about the best method for closing the abdominal wall after AAA repair surgery is investigated in this study. Even though the use of mesh has been demonstrated to significantly lower the incidence of incisional hernias, worries about seroma formation and potential infection risk continue to be a major obstacle to its widespread use. Due in large part to the possible severity of mesh-related infections, these worries continue even though data do not indicate a statistically significant difference in infection rates between the mesh and no mesh groups. Problems like the development of an abscess or the requirement to remove the mesh can lead to longer recovery periods, higher medical expenses, and worse patient outcomes.

Additionally, surgeons frequently take a cautious approach due to the uncertainty surrounding the clinical evidence, which includes the large confidence intervals and the small number of infection events. The choice of closure technique is made more complex by patient-specific factors, such as the risk of ischaemia, underlying comorbidities, and whether the AAA repair is done in an emergency or elective setting. By tackling these problems and analysing the advantages and disadvantages of using mesh, this study provides insightful information to guide surgical decision-making. The results of this review show a clear and statistically significant benefit of using mesh reinforcement in reducing the risk of incisional hernias following AAA repair, even in the presence of moderate heterogeneity across studies. While the mesh group showed a higher incidence of seroma, this complication is typically mild and self-limiting and far less serious than an incisional hernia. Infection rates were low and did not differ significantly between groups. These results support the use of prophylactic mesh as an effective and safe approach to preventing incisional hernia in appropriately selected patients.

Limitations

While this study has several strengths, it is essential to acknowledge its limitations. The included RCTs exhibit inherent variability, potentially introducing heterogeneity into the meta-analysis and thereby impacting the reliability of the findings. Also, there is the possibility of publication bias, wherein studies with favourable outcomes are more likely to be published, potentially skewing the overall results. The generalisability of the findings may be constrained by variances in patient demographics, surgical methodologies, and healthcare contexts across the diverse array of studies. A risk of bias chart (Table 4) illustrates this in greater detail across the different studies. Moreover, the decision about mesh use may be greatly impacted by the inclusion of patients with AAA in both emergency and elective settings. This decision-making process is further complicated by the need to take the risk of organ ischaemia into account, which could have a significant impact on the choice of mesh because of possible postoperative infection issues. The principal objection to the use of mesh in initial repair is the possibility of infection and the consequent need to remove the mesh. This point emphasises the delicate balancing that must be done to choose the best surgical strategy for AAA repair, considering both the immediate results of the procedure and potential long-term consequences.

Further Studies

Future research in this area should focus on addressing the remaining uncertainties and expanding our understanding of the optimal use of mesh in abdominal wall closure following AAA repair surgery. Large-scale, multicentre RCTs with standardised protocols are essential to validate the findings of this meta-analysis and provide high-quality evidence for clinical practice. Comparative effectiveness studies evaluating different types of mesh and placement techniques are warranted to optimise patient outcomes and minimise healthcare costs.

## Conclusions

While mesh reinforcement shows promise in reducing the incidence of incisional hernias following AAA repair surgery, it is associated with increased seroma formation. Clinicians should consider the available evidence in conjunction with patient-specific factors when making decisions regarding abdominal wall closure techniques. Further research is needed to refine surgical practices and optimise patient outcomes in the management of AAA repair surgeries.

## References

[1] (2024). Abdominal aortic aneurysm (AAA). https://www.ulh.nhs.uk/services/abdominal-aortic-aneurysm.

[2] Song P, He Y, Adeloye D (2023). The global and regional prevalence of abdominal aortic aneurysms: a systematic review and modeling analysis. Ann Surg.

[3] (2025). UK AAA screening programmes: 10-year effectiveness review. https://www.gov.uk/government/publications/aaa-screening-programmes-in-the-uk-10-year-effectiveness-review/uk-aaa-screening-programmes-10-year-effectiveness-review.

[4] Barranquero AG, Molina JM, Gonzalez-Hidalgo C (2022). Incidence and risk factors for incisional hernia after open abdominal aortic aneurysm repair. Cir Esp (Engl Ed).

[5] Smith L, Wilkes E, Rolfe C, Westlake P, Cornish J, Brooks P, Torkington J (2024). Incidence, healthcare resource use and costs associated with incisional hernia repair. J Abdom Wall Surg.

[6] Endo T, Miyahara K, Shirasu T, Mochizuki Y, Taniguchi R, Takayama T, Hoshina K (2023). Risk factors for incisional hernia after open abdominal aortic aneurysm repair. In Vivo.

[7] De Paulis S, Arlotta G, Calabrese M (2022). Postoperative intensive care management of aortic repair. J Pers Med.

[8] Ahmed J, Hasnain N, Fatima I (2020). Prophylactic mesh placement for the prevention of incisional hernia in high-risk patients after abdominal surgery: a systematic review and meta-analysis. Cureus.

[9] Durbin B, Spencer A, Briese A, Edgerton C, Hope WW (2023). If evidence is in favor of incisional hernia prevention with mesh, why is it not implemented?. J Abdom Wall Surg.

[10] Woolson RF, Bean JA (1982). Mantel-Haenszel statistics and direct standardization. Stat Med.

[11] Bali C, Papakostas J, Georgiou G (2015). A comparative study of sutured versus bovine pericardium mesh abdominal closure after open abdominal aortic aneurysm repair. Hernia.

[12] Bevis PM, Windhaber RA, Lear PA, Poskitt KR, Earnshaw JJ, Mitchell DC (2010). Randomized clinical trial of mesh versus sutured wound closure after open abdominal aortic aneurysm surgery. Br J Surg.

[13] Dewulf M, Muysoms F, Vierendeels T (2022). Prevention of incisional hernias by prophylactic mesh-augmented reinforcement of midline laparotomies for abdominal aortic aneurysm treatment: five-year follow-up of a randomized controlled trial. Ann Surg.

[14] Honig S, Diener H, Kölbel T, Reinpold W, Zapf A, Bibiza-Freiwald E, Debus ES (2022). Abdominal incision defect following AAA-surgery (AIDA): 2-year results of prophylactic onlay-mesh augmentation in a multicentre, double-blind, randomised controlled trial. Updates Surg.

[15] Jairam AP, Timmermans L, Eker HH (2017). Prevention of incisional hernia with prophylactic onlay and sublay mesh reinforcement versus primary suture only in midline laparotomies (PRIMA): 2-year follow-up of a multicentre, double-blind, randomised controlled trial. Lancet.

[16] Higgins JP, Altman DG, Gøtzsche PC (2011). The Cochrane Collaboration's tool for assessing risk of bias in randomised trials. BMJ.

[17] Nieuwenhuizen J, Eker HH, Timmermans L, Hop WC, Kleinrensink GJ, Jeekel J, Lange JF (2013). A double blind randomized controlled trial comparing primary suture closure with mesh augmented closure to reduce incisional hernia incidence. BMC Surg.

